# Transcriptome sequencing reveals the effect of biochar improvement on the development of tobacco plants before and after topping

**DOI:** 10.1371/journal.pone.0224556

**Published:** 2019-10-31

**Authors:** Shen Yan, Zhengyang Niu, Haitao Yan, Aigai Zhang, Guoshun Liu

**Affiliations:** 1 Department of Tobacco cultivation, College of Tobacco Science, Henan Agricultural University, Zhengzhou, Henan Province, China; 2 Henan Biochar Engineering Technology Research Center, Zhengzhou, Henan Province, China; 3 Henan Biochar Technology Engineering Laboratory, Zhengzhou, Henan Province, China; 4 Department of Microbiology, College of Agriculture and Life Science, Cornell University, Ithaca, NY, United States of America; RMIT University, AUSTRALIA

## Abstract

The application of biochar is one of the most useful methods for improving soil quality, which is of the utmost significance for the continuous production of crops. As there are no conclusive studies on the specific effects of biochar application on tobacco quality, this study aimed to improve the yield and quality of tobacco as a model crop for economic and genetic research in southern China, by such application. We used transcriptome sequencing to reveal the effects of applied biochar on tobacco development before and after topping. Our results showed that topping affected carbon and nitrogen metabolism, photosynthesis and secondary metabolism in the tobacco plants, while straw biochar-application to the soil resulted in amino acid and lipid synthesis; additionally, it affected secondary metabolism of the tobacco plants through carbon restoration and hormonal action, before and after topping. In addition to the new insights into the impact of biochar on crops, our findings provide a basis for biochar application measures in tobacco and other crops.

## Introduction

China has a long history of plant cultivation and is one of the countries with the highest agricultural production output [1]. In addition to cereal grains per se, great amounts of straw are produced every year, which account for nearly one-third of the global production of crop residues [2]. As crop production has dramatically increased over the past few decades, people have begun to search for new ways to use this abundant resource [3]. Some of the crop straw returns to the soil through soil management practice [4]; however, owing to its limited extent, such return has little impact [1, 5]; further, straw promotes pests, causes short-term soil acidification, and would be an inconvenience for field management if it is returned to the fields in excessive amounts [6, 7]. Additionally, burning of crop straw in the field after harvest, as in most cropping areas of southern China [8], has led to severe environmental and human health problems [9–11]. Therefore, it is urgent for China to find a high-efficiency, cost-effective, and environment-friendly strategy for the utilization of residual crop straw. Recently, biochar has become increasingly popular in China, as it gives farmers an excellent alternative to utilize crop straw resources.

Biochar is a carbon-rich by-product that results from pyrolysis of biomass at high temperature and low-oxygen tension during biofuel production [12]. Previous research showed that biochar promoted crop growth [13]. Biochar influences a wide range of soil properties [14–16], such as soil water-holding capacity, soil carbon and nitrogen cycling, microbial community, bulk density, and soil pH; all due to its porous structure, high surface area, and affinity for charged particles [17]. Biochar can interact with the soil and can have cascading effects throughout the ecosystem [18, 19], thereby affecting plant growth profoundly. Therefore, biochar is a by-product of bioenergy production that shows a high potential for increasing crop productivity while reducing the use of fertilizer significantly. Biochar has been frequently touted as a “win-win” solution to global environmental challenges [20, 21]. However, large differences in the response of plants and soils to biochar have been reported, which may also lead to uncertainty as to the beneficial interaction between biochar and plants [12].

Flue-cured tobacco is an important economic commodity in China. The removal of the tobacco inflorescence, together with a few of the youngest leaves–known as tobacco topping–is performed to prevent reproductive organs and young leaves from competing with the valuable older leaves for nutrients. Studies have shown that tobacco topping significantly affected the growth and development of the plant, not only root growth [22, 23], and leaf quality [24]; additionally, topping alters many biological processes [25]. Therefore, analyzing plant quality before and after topping is a high-priority in research. In recent years, some studies have shown that applied biochar might improve the -grwoth of tobacco leaves [26]; however, the underlying mechanism is not yet clear, especially considering that the development of the tobacco plant is divided into two stages by topping.

Therefore, in this study we investigated the effects of topping and biochar application to the soil on transcriptional modifications during development of the tobacco plant. The results showed that topping affected carbon and nitrogen metabolism, photosynthesis and secondary metabolic processes in tobacco leaves, while biochar applied to the soil affected amino acid and lipid synthesis, as well as secondary metabolism before and after topping through carbon metabolism and hormone action. Overall, these data provide novel and valuable information that will surely help in understanding the impact of straw biochar on the mechanisms that regulate crop development, while providing guidance for improving straw biochar management for tobacco production.

## Materials and methods

### Plant materials and growth conditions

The test site is located in the tobacco production fields of Xiniu Town, Xinfeng County, Ganzhou City (25° 27′ 11.71″ N; 114° 51′ 54.25″ E). The study was carried out on private land and the owner gave permission to conduct the study on this site. The area is characterized by a subtropical, temperate monsoon climate with annual sunshine ranging from 1473.3 to 2077.5 h. The average annual temperature is 18–19.7°C, the average frost-free period is 250 d, and the annual rainfall ranges from 1410 to 1762 mm. The soil at the site is a typical high-yielding paddy soil classified as a hydroagric Stagnic Anthrosol [27] and an Entic Halpudept [28]. Soil characteristics at the site are summarized in Table 1.

**Table 1:** Nutrient status of the experimental soils.

Soil type	Organic matter (mg·g^−1^)	Hydro-N (mg·kg^−1^)	Available P (mg·kg^−1^)	Available K (mg·kg^−1^)	pH	Total C (mg·g^−1^)	Total N (mg·g^−1^)
Paddy soil	27.04	129.8041	24.1967	109.513	5.59	16.6	2.1

A biochar application experiment was conducted in these paddy soils early in March 2017. At this time, 25 kg of the topsoil layer (0–20 cm) was placed in plastic pots and left standing in the open for seven days. Next, control (CK) and treatment (T) pots received 0 or 40 g biochar pot^-1^, respectively. The biochar used in this study was pyrolyzed from wheat straw in a vertical kiln at a temperature of 350–550°C at Sanli New Energy Company Henan, China. The characteristics of the biochar used are listed in S1 Data. Nitrogen was applied equally to all pots at a rate of 7 g pot^−1^ for a final N:P:K ratio of 1:0.9:2.8, as per local planting management guidelines. Subsequently, tobacco (*Nicotiana tabacum* ‘Yunyan 87’) seedlings were transplanted into the aforementioned pots at a density of one seedling per pot; 25 pots of each treatment were included. Management of the plant material during the experimental period followed local common practice. Information on the growth of the tobacco plants can be found in S2 and S3 Data.

### Sample collection

As previously described [29], samples were collected before and after topping, 60 and 70 d after planting, respectively. In order to avoid the influence of sampling between the two sampling time-points, different plants were chosen on each occasion. Three representative tobacco plants were selected for each replication, and the 12th leaf counted from the bottom of the plants was harvested; leaves between the 6–7 veins were sampled and the three leaves were mixed into single samples that were immediately placed in liquid nitrogen and stored at –80°C for RNA extraction. Each treatment was sampled in triplicate.

### cDNA library preparation and transcriptome sequencing

Total RNA was extracted using trizol [30]. Integrity of extracted RNA was verified by RNase free agarose gel electrophoresis and the concentration was measured using a 2100 Bioanalyzer (Agilent Technologies, USA). High-quality RNA was used for subsequent sequencing. A cDNA library was constructed using NEB Next Ultra RNA Library Prep Kit for Illumina (New England Biolabs, USA) following the protocol recommended by the manufacturer [31]. In brief, mRNA was enriched by Oligo (dT) beads; next, the enriched mRNA was fragmented into short fragments using fragmentation buffer and then reverse transcribed into cDNA with random primers. Second-strand cDNA was synthesized by DNA polymerase I, RNase H, dNTP and buffer. Then the cDNA fragments were purified, end repaired, poly(A) tailed, and ligated to Illumina sequencing adapters. The ligation products were selected by size using agarose gel electrophoresis, amplified by PCR, and finally sequenced using Illumina HiSeqTM 4000 (Gene Denovo Biotechnology Co., Guangzhou, China).

High quality clean reads were obtained by further filtering reads to remove: 1) reads containing adapters; 2) reads containing more than 10% of unknown nucleotides (N); 3) low-quality reads containing more than 50% of low-quality bases (Q-value ≤20).

### Transcript reconstruction

The reconstruction of transcripts was carried out with Cufflinks software [32], which together with TopHat2, allows biologists to identify new genes and new splice variants of known ones. The reference annotation-based transcripts (RABT) program was preferred [33]. Cufflinks constructed faux reads according to reference to make up for the influence of low coverage sequencing. During the last step of assembly, all the reassembled fragments were aligned with reference genes and then similar fragments were removed. Next, we used Cuffmerge to merge transcripts from different replicas of a group into a comprehensive set of transcripts, and then transcripts from multiple groups were merged into a finally comprehensive set of transcripts for further downstream differential expression analysis.

### Quantification of gene abundance

Gene abundances were quantified by RSEM software [34]. Two steps were followed for RSEM to quantify gene abundances. Firstly, a set of reference transcript sequences were generated and preprocessed according to known transcripts, new transcripts (in FASTA format), and gene annotation files (in GTF format). Secondly, RNA-seq reads were re-aligned to the reference transcripts using the Bowtie alignment program and the resulting alignments were used to estimate gene abundances. Gene expression levels were normalized by using the FPKM (Fragments Per Kilobase of transcript per Million mapped reads) method.

### qRT-PCR verification

Four representative genes in key pathways of straw biochar applied were selected for quantitative RT-PCR analysis; the method was performed using a procedure described previously [35]. We compared the expression of representative genes and the relative expression of different genes by one-way ANOVA and Tukey test comparisons. Statistical significance was set to P < 0.05.

### Principal component analysis

Principal component analysis (PCA) was performed with R package g-models (http://www.r-project.org/).

### Differentially expressed genes (DEGs) analysis

The edgeR package (http://www.rproject.org/) was used to identify differentially expressed genes across samples or groups [36]. Genes with a fold change ≥ 2 and a false discovery rate (FDR) <0.05 in a comparison were identified as significant DEGs, which were then subjected to enrichment analysis of GO functions and KEGG pathway [37].

### Weighted gene co-expression network analysis

Co-expression networks were constructed using WGCNA (v1.47) package in R[38].

### Data deposition

The transcriptome sequence data obtained in this study can be found at the National Center for Biotechnology Information (NCBI) Sequence Read Archive under accession numbers: PRJNA448573.

## Results

### Sequencing quality and sample relationships

Twelve samples were sequenced to obtain 750,347,666 reads, a total of 112.6 Gb of data. After filtering, 741,735,220 high quality clean reads were retained, a total of 111.0 Gb data (S4 Data). A total of 9,827,196 multiple mapped reads were obtained by comparing to the reference genomes after removing ribosomal RNAs. The sequencing saturation curves for each sample showed that the sequencing depth met the requirements for subsequent analysis (S5 Data). In all, 55,343 genes were detected across samples, which accounted for 74.70% of the total number of genes (74,088) in the reference group. In addition, 2,170 new genes that were not found in the reference groups were also detected, reflecting the genetic differences between the experimental materials and reference tobacco. These new genes were assembled and annotated, and are shown in S6 Data.

**Fig 1:**
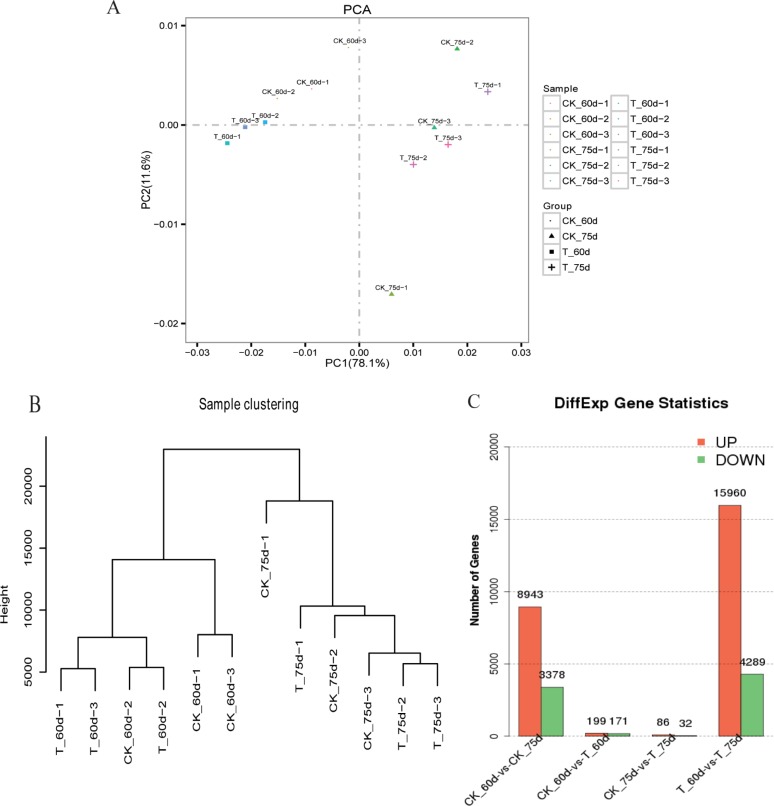
Differences in transcriptome sequencing between control and treatment groups before and after topping. A) Sample component analysis; B) Sample clustering; C) Difference in the number of genes significantly up-/down-regulated between samples; the x-axis shows the paired samples; the y-axis shows the number of DEGs; red bars represent significantly upregulated genes; green bars represent significantly downregulated genes.

PCA analysis and clustering of samples were performed based on gene expression level. The results of PCA analysis showed that the differences between treatment and control groups was more pronounced at 60 days; cluster analysis confirmed these results (Fig 1A and 1B). The correlation test between different replicates showed that the gene expression patterns were consistent across the three samples from different treatment periods (S7 Data). Differential gene analysis during different processing times showed that there were relatively few DEGs between the two treatments, and that there were more DEGs before than after topping. Further, there were more DEGs at different times, and there were more DEGs in the treated groups (Fig 1C). This showed that tobacco plants experienced significant changes before and after topping. Although the use of soil organic carbon may continue to affect the growth and development of tobacco plants, its effects were more pronounced before topping.

### Intrinsic biological changes in tobacco from pre-topping (60 DAP) to post-topping (75 DAP) under different conditions

**Fig 2:**
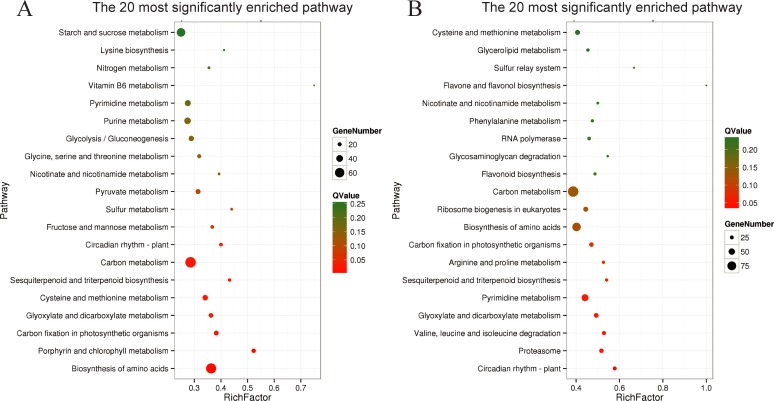
Pathway enrichment of differentially expressed genes before and after topping under different conditions. A) KEGG enrichment map of differentially expressed genes between CK_60 d and CK_75 d; B) KEGG enrichment map of differentially expressed genes between BT_60 d and BT_75 d. RichFactor refers to the ratio of the number of genes in the differentially expressed genes located in the pathway entry to the total number of genes in the pathway entry in all genes. The larger the RichFactor, the higher the degree of enrichment. Q-Value is the P-Value after multiple hypothesis tests and corrections. The value ranges from 0 to 1. The closer to zero, the more significant the enrichment. The map is plotted using the top 20 pathways of Q-Values from the smallest to the largest.

Under control conditions, there were 12,321 differentially expressed genes in the 75-d samples, compared to the 60-d samples. Among these, 8,943 were upregulated, while 3,378 were downregulated (S8 Data). These genes were then employed for KEGG enrichment analysis. Ten representative pathways were among the most significantly enriched, including ko01230 (biosynthesis of amino acids), ko00860 (porphyrin and chlorophyll metabolism), ko00710 (carbon fixation in photosynthetic organisms), ko01200 (carbon metabolism), ko00909 (sesquiterpenoid and triterpenoid biosynthesis), ko00270 (cysteine and methionine metabolism), ko00630 (glyoxylate and dicarboxylate metabolism), ko04712 (circadian rhythm), ko00051 (fructose and mannose metabolism), and ko00920 (sulfur metabolism) (Fig 2A).

In the case of biochar application, there were 20,249 differentially expressed genes in the 75-d samples, compared to the 60-d samples; among them, 15,960 were upregulated and 4,289 were downregulated (S8 Data). These genes were then employed for KEGG enrichment analysis. Ten representative pathways were among the most significantly enriched, including ko04712 (circadian rhythm), ko03050 (proteasome), ko00280 (valine, leucine and isoleucine degradation), ko00630 (glyoxylate and dicarboxylate metabolism), ko00240 (pyrimidine metabolism), ko00909 (sesquiterpenoid and triterpenoid biosynthesis), ko00710 (carbon fixation in photosynthetic organisms), ko00330 (arginine and proline metabolism), ko01230 (biosynthesis of amino acids), and ko03008 (ribosome biogenesis in eukaryotes) (Fig 2B).

By comparison, we found that some pathways were highly enriched under both, control and treatment conditions, such as the biosynthesis of amino acids, porphyrin and chlorophyll metabolism, carbon fixation in photosynthetic organisms, carbon metabolism, sesquiterpenoid and triterpenoid biosynthesis, glyoxylate and dicarboxylate metabolism, circadian rhythm, and pyrimidine metabolism, among others. This showed that, during the time interval between 60 and 75 DAP, i.e., before and after topping, respectively, tobacco plants underwent dramatic biological changes in terms of carbon metabolism, nitrogen metabolism, photosynthesis, and terpenoid synthesis, among other pathways. Other pathways were only significantly enriched in control or treatment groups, such as cysteine and methionine metabolism, fructose and mannose metabolism, sulfur metabolism, proteasome, valine, leucine and isoleucine degradation, arginine and proline metabolism and ribosome biogenesis in eukaryotes. As can be seen, protein and amino acid metabolism were significantly altered by the application of biochar before and after topping.

### Application of biochar affected the metabolism of tobacco leaves before and after topping

**Fig 3:**
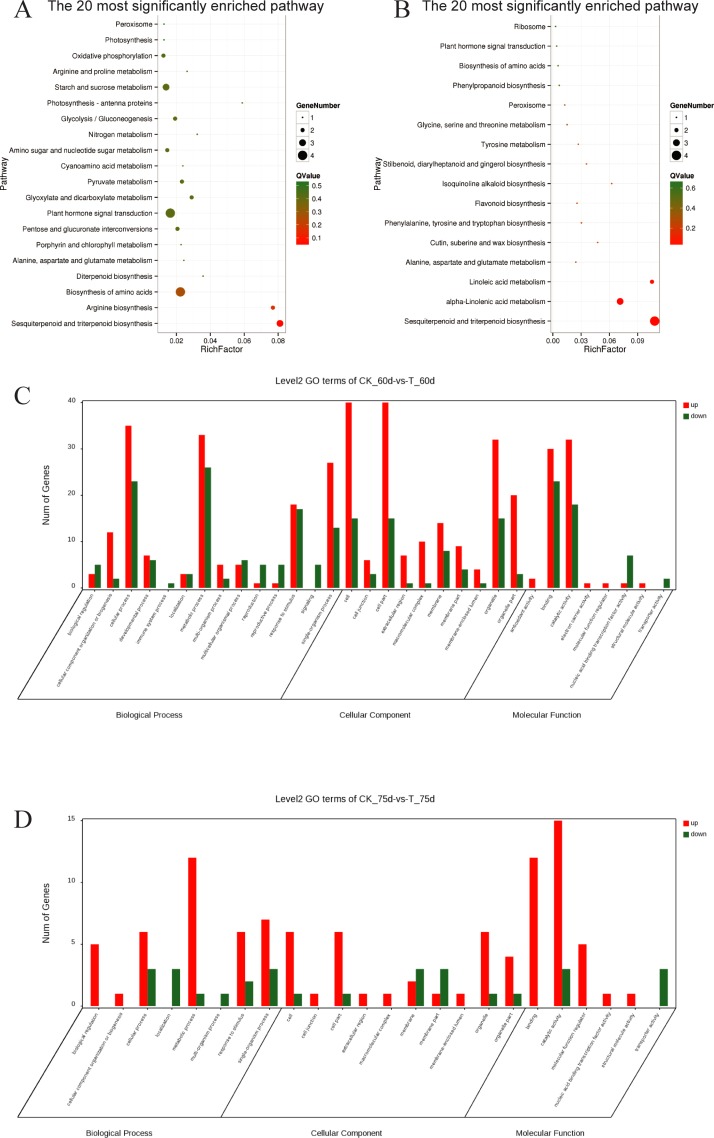
Biological processes involved in differentially expressed genes in control and treatment groups before and after topping. A) KEGG enrichment map of differentially expressed genes for CK_60 d and T_60 d; B) KEGG enrichment map of differentially expressed genes for CK_75 d and T_75 d; C) GO classification chart of CK_60 d and T_60 d differentially expressed genes; D) GO classification chart of CK_75 d and T_75 d differentially expressed genes. RichFactor refers to the ratio of the number of genes in the differentially expressed genes located in the pathway entry to the total number of genes in the pathway entry in all genes. The larger the RichFactor, the higher the degree of enrichment. Q-Value is the P-Value after multiple hypothesis tests and corrections. The value ranges from 0 to 1. The closer to zero, the more significant the enrichment. The map is plotted using the top 20 pathways of Q-values from the smallest to the largest.

There were 370 DEGs in the treated group compared to the control, among 60-d samples; among these, 199 were upregulated and 171 were downregulated (S8 Data). These genes were then employed for KEGG enrichment analysis. Three representative pathways were significantly enriched: ko00909 (sesquiterpenoid and triterpenoid biosynthesis), ko00220 (arginine biosynthesis) and ko01230 (biosynthesis of amino acids) (Fig 3A). These findings indicated that before topping, the impact of straw biochar applied was mainly reflected in the process of amino acid synthesis.

There were 118 DEGs in the treatment group compared to the control group in 75-d samples; among these, 86 were upregulated and 32 were downregulated (S8 Data). These genes were then employed for KEGG enrichment analysis. Four representative pathways were significantly enriched: ko00909 (sesquiterpenoid and triterpenoid biosynthesis), ko00592 (alpha-linolenic acid metabolism), ko00591 (linoleic acid metabolism), and ko00950 (isoquinoline alkaloid biosynthesis) (Fig 3B). These results indicated that after topping, the impact of straw biochar applied was mainly reflected in the GO classification of these differentially expressed genes. Straw biochar applied led to significant changes in biological metabolism regardless of topping (Fig 3C and 3D). Interestingly, differentially expressed genes were significantly enriched in the sesquiterpenoid and triterpenoid biosynthesis process at either 60 d or 75 d in both, control and treatment groups. However, at 60 d, differentially expressed genes were enriched in the process of sesquiterpenoid synthesis, whereas at 75 d, they were enriched in the process of triterpenoid synthesis. This suggests a complex relationship between terpenoid metabolism and applied straw biochar.

### Weighted gene co-expression network analysis revealed gene feature differences between biochar-treated and control groups at different stages

**Fig 4:**
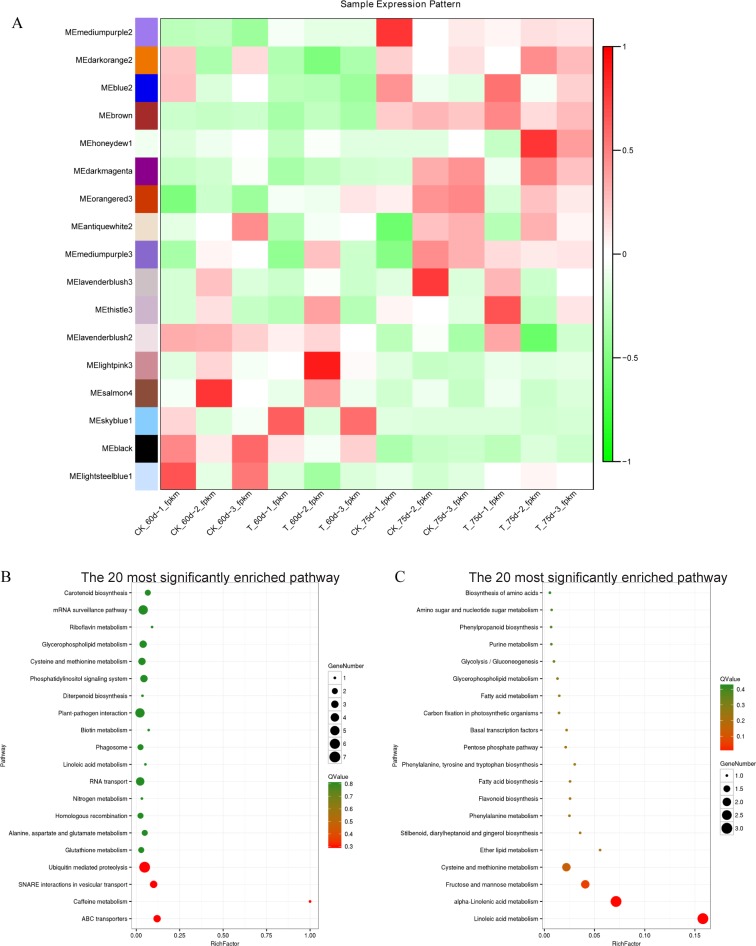
Functional analysis of key expression patterns of WGCNA. A) Expression patterns of different modules of WGCNA; B) KO enrichment bubble diagram of genes in darkorange2 module; C) KO enrichment bubble diagram of genes in honeydew1 module.

Due to the large number of samples analyzed by transcriptome sequencing, we classified the differentially expressed genes by weighted gene co-expression network analysis (WGCNA) (Fig 4A). All differentially expressed genes were classified into 17 modules. Based on the gene expression pattern of these modules, we focused on the following two of the feature ones.

Overall, the expression of the darkorange2 module was lower in the treated than in the control group before topping, while it was higher in the treated than in the control group after topping. KEGG enrichment of the DEGs in this module revealed that the processes related to plant hormone signal transduction and carbohydrate metabolism were significantly enriched (Fig 4B). This result confirmed that the application of biochar affected the metabolism of carbohydrates in tobacco plants at different times. By mapping the genes involved in hormone signal transduction, we found that the application of biochar altered the regulation of abscisic acid, ethylene, jasmonic acid, and salicylate hormones (S9 Data).

The overall expression of the honeydew1 module was not significantly different between the two groups before topping, and it was significantly higher in the treatment group than in the control group after topping. KEGG enrichment of differentially expressed genes in this module revealed significant enrichment of processes such as linoleic acid metabolism, alpha-linolenic acid metabolism, fructose and mannose metabolism, cysteine and methionine metabolism, and ether lipid metabolism (Fig 4C). This showed that after the use of biochar, metabolic processes of lipid metabolism changed significantly after topping.

**Fig 5:**
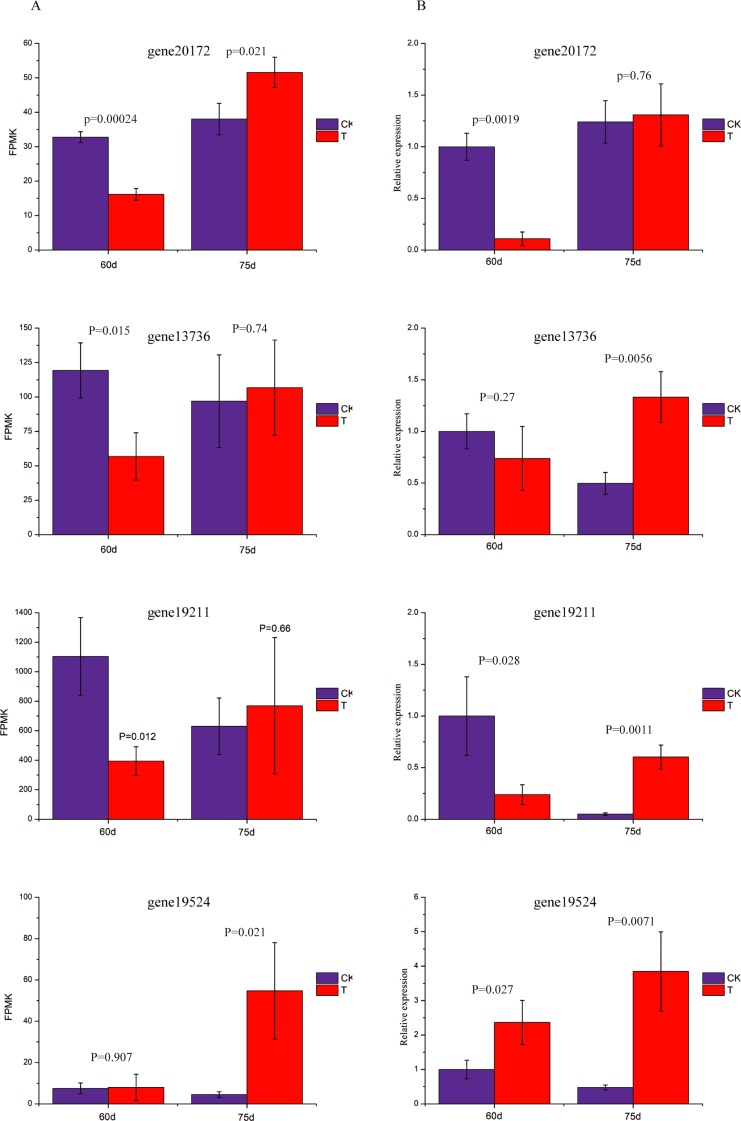
qRT-PCR validation of representative genes in key pathways affected by straw biochar application. A) Expression of representative genes in transcriptome data; B) Relative expression level of representative genes detected by qRT-PCR.

To verify the reliability of these results, we selected genes 20,172 and 13,736 in hormone transduction and carbon metabolism, respectively, and 19,211 and 195,195 in alpha-linolenic acid metabolism, and verified with qRT-PCR. We found that the differences in expression of these genes between treatment and control groups matched the expression patterns in the corresponding modules (S10 Data). However, there were differences in the fold changes, which may indicate the difference in sensitivity between the two methods (Fig 5).

## Discussion

### Topping altered the normal development of tobacco plants

Topping blocked the passage of tobacco plants from vegetative to reproductive growth, thus causing changes in normal development. Previous studies have shown that topping shifts the circulation of raw materials within tobacco plants from nitrogen- to carbon metabolism-related pathways, thereby affecting photosynthesis and secondary metabolic processes [22, 39]. Our study confirmed these effects at the molecular level. The differentially expressed genes before and after topping were enriched in metabolic processes related to the biosynthesis of amino acids, carbon metabolism, glyoxylate and dicarboxylate metabolism, and pyrimidine metabolism, regardless of biochar treatment. These results were consistent with previous studies in that topping significantly affected nitrogen and carbon metabolism in tobacco plants [29, 40]. Porphyrin and chlorophyll metabolism, carbon fixation in photosynthetic organisms, and sesquiterpenoid and triterpenoid biosynthesis processes were also significantly enriched before and after topping, which verified previous findings on the effects of topping on photosynthesis and secondary metabolism of tobacco plants [40].

### Application of straw biochar altered the normal development of tobacco plants

We detected 370 differentially expressed genes between control and straw-biochar-treated groups before topping. These genes were significantly enriched in the processes related to amino acid biosynthesis. This observation indicated that straw biochar applied before topping affected the process of amino acid synthesis. Based on previous studies, the main intrinsic metabolism in tobacco, prior to topping, is nitrogen metabolism; therefore, straw biochar applied before topping may have important influence on tobacco development [41].

On the other hand, we detected 118 differentially expressed genes between the control and straw-biochar-treated groups after topping. These genes were significantly enriched in the processes of sesquiterpenoid and triterpenoid biosynthesis, alpha-linolenic acid metabolism, linoleic acid metabolism and isoquinoline alkaloid biosynthesis, which pertain to lipid and secondary metabolism. In addition, the genes that did not differ before topping but significantly increased after topping in the biochar-treated group were also significantly enriched in lipid-related pathways, as shown by WGCNA analysis. These results suggested that straw biochar treatment affected the development of tobacco flavor after topping, which is also consistent with previous studies [41, 42].

According this analysis, the difference in development between control and biochar-treated tobacco plants as a result of biochar application is likely related to carbon and hormonal metabolism, including abscisic acid, ethylene, jasmonic acid, and salicylic acid.

### Application organic carbon to the soil effectively improved the quality of tobacco

Our results showed that the application of organic carbon to the soil produced significant effects on normal development of tobacco plants. Before topping, it mainly affected nitrogen metabolism, while after topping, it affected lipid and secondary metabolism. Genes related to carbon metabolism were downregulated before topping and upregulated after topping. The mechanism of regulation during the critical period of carbon metabolism seems sophisticated and complex, and must be further investigated.

The results reported herein explained the mechanisms underlying our previous findings. In a previous study, we found that the application of soil organic carbon significantly improved the yield and the flavor of tobacco leaves. The neutral aroma content after baking was similar to that of high-quality tobacco leaves. The aroma quality and quantity, aftertaste, and customer feedback were significantly higher than for other flue-cured tobacco, while miscellaneous irritating smokes were significantly lower [41, 43].

Altogether, our results showed that topping affected carbon and nitrogen metabolism, photosynthesis and secondary metabolism in the tobacco plant, while straw biochar-application to the soil resulted in enhanced amino acid and lipid synthesis and affected secondary metabolism before and after topping. The amino acids can react with sugars in a Maillard reaction, which is one of the important reactions in the formation of the characteristic aroma of tobacco, and its product is an important aroma precursor [44]. The enhancement of lipid metabolism may affect the aroma and taste of tobacco leaves [45]. These results provide new insights into the molecular mechanisms for improvement of plant performance by the application of organic carbon to the soil.

## Supporting information

S1 Data Characteristics of the biochar applied to the experimental pots. (DOCX)

S2 Data Photograph of biochar treated (T) and untreated (CK) tobacco plants. (JPG)

S3 Data Growth data for biochar treated and untreated tobacco plants. (PDF)

S4 Data Summary of the RNA-seq results of tobacco leaves. (XLSX)

S5 Data Sequencing saturation curves for each sample. (PDF)

S6 Data New genes in tobacco leaves. (TXT)

S7 Data Correlation analysis among different replicates. (PDF)

S8 Data Information of differentially expressed genes. (XLSX)

S9 Data Differentially expressed genes involved in the plant hormone signal transduction pathway in tobacco. (PNG)

S10 Data Correlative statistic for the gene between KEGG and qRT-PCR expressed genes. (DOCX)
