# Supplementary material for: Transcriptome sequencing reveals the effect of biochar improvement on the development of tobacco plants before and after topping
Source: PLoS One. 2019 Oct 31;14(10):e0224556. doi: 10.1371/journal.pone.0224556 (PMC6822942; doi:10.1371/journal.pone.0224556)
Supplement: S1 Data — (DOCX) [file pone.0224556.s001.docx]

Supplementary data 1 Characteristics of the biochar applied to the experimental plots.

| Variable | Biochar |
| --- | --- |
| Total C (%) | 52.8 |
| Total N (%) | 0.41 |
| pH | 9.36 |
| Ash (%) | 11.2 |
| Specific surface area（m^2^ g^-1^） | 16.7 |
| Nitrate-N (mg kg^-1^) | 0.38 |
| Aluminum (%) | 0.17 |
| Arsenic (mg kg^-1^) | <4 |
| Boron (mg kg^-1^) | 13 |
| Calcium (%) | 5.6 |
| Cadmium (mg kg^-1^) | <0.15 |
| Cobalt (mg kg^-1^) | 4.3 |
| Chromium (mg kg^-1^) | 7.9 |
| Copper (mg kg^-1^) | 26 |
| Iron (%) | 0.5 |
| Potassium (%) | 0.41 |
| Magnesium (%) | 1.22 |
| Manganese (mg kg^-1^) | 1893 |
| Molybdenum (mg kg^-1^) | 1.8 |
| Sodium (%) | 0.16 |
| Nickel (mg kg^-1^) | 5.8 |
| Phosphorus (%) | 2.3 |
| Lead (mg kg^-1^) | 3.7 |
| Sulfur (%) | 0.39 |
| Selenium (mg kg^-1^) | <3 |
| Zinc (mg kg^-1^) | 117 |
