# Supplementary figures and images for: Transcriptome sequencing reveals the effect of biochar improvement on the development of tobacco plants before and after topping
Source: PLoS One. 2019 Oct 31;14(10):e0224556. doi: 10.1371/journal.pone.0224556 (PMC6822942; doi:10.1371/journal.pone.0224556)

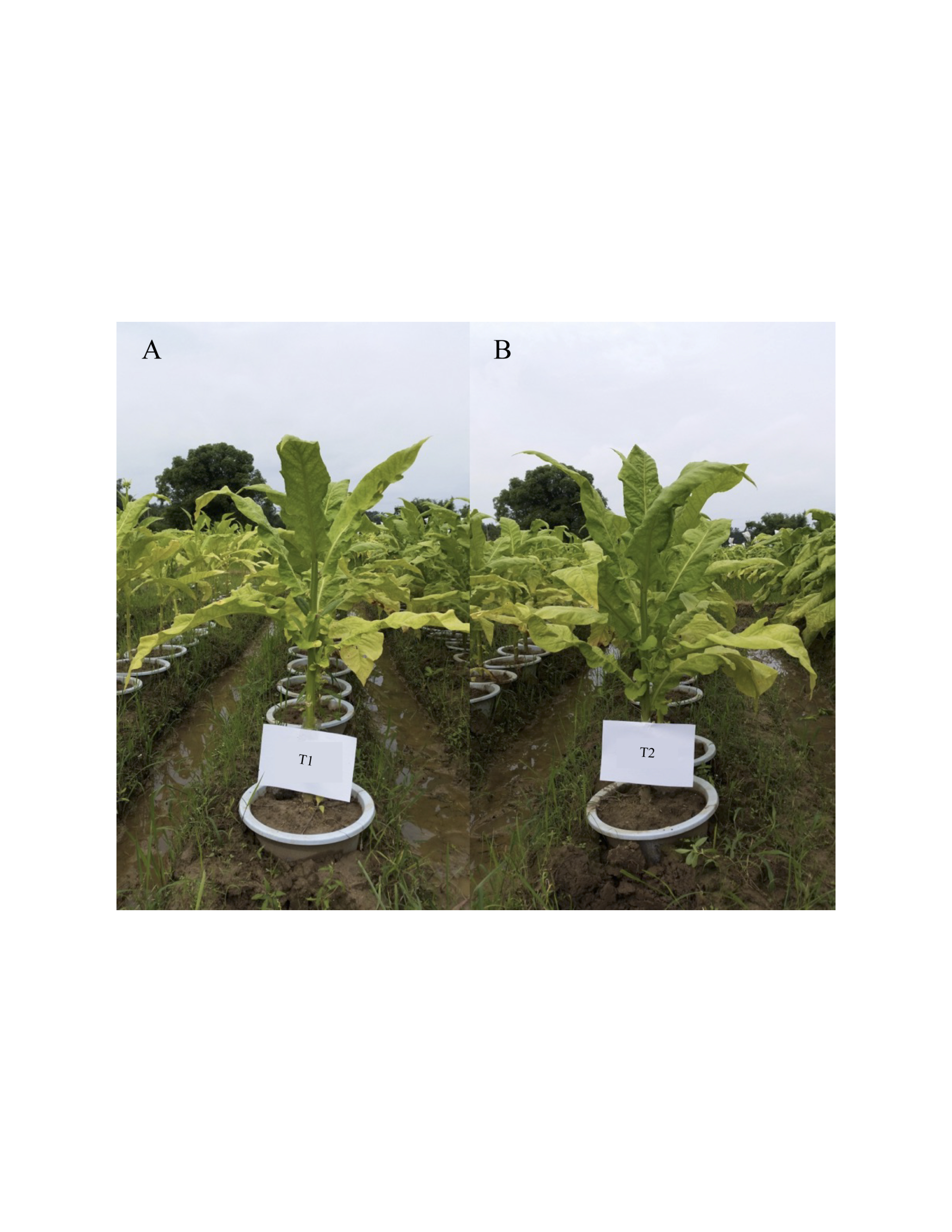

Supplement: S2 Data — (JPG) [file pone.0224556.s002.jpg]

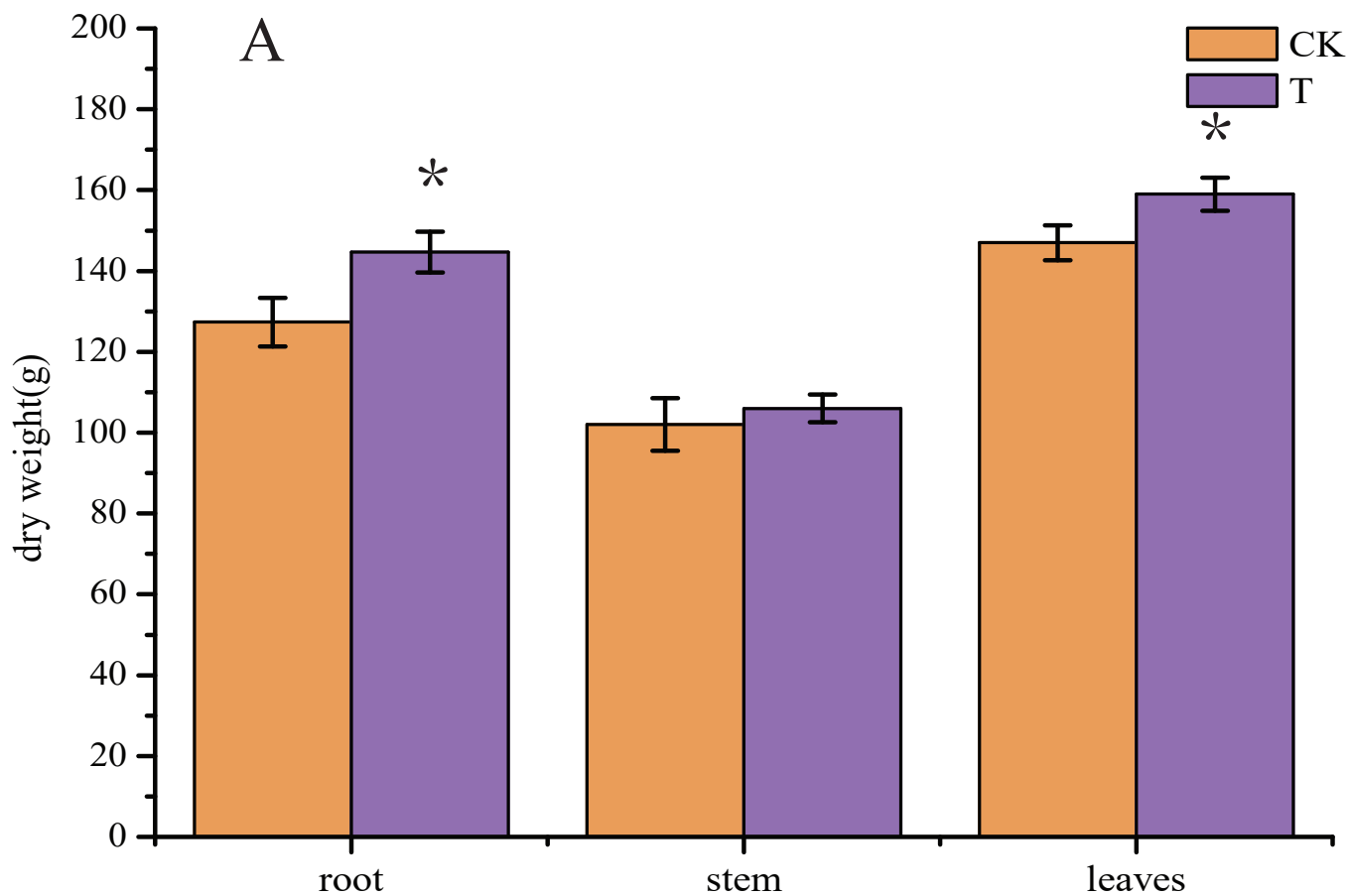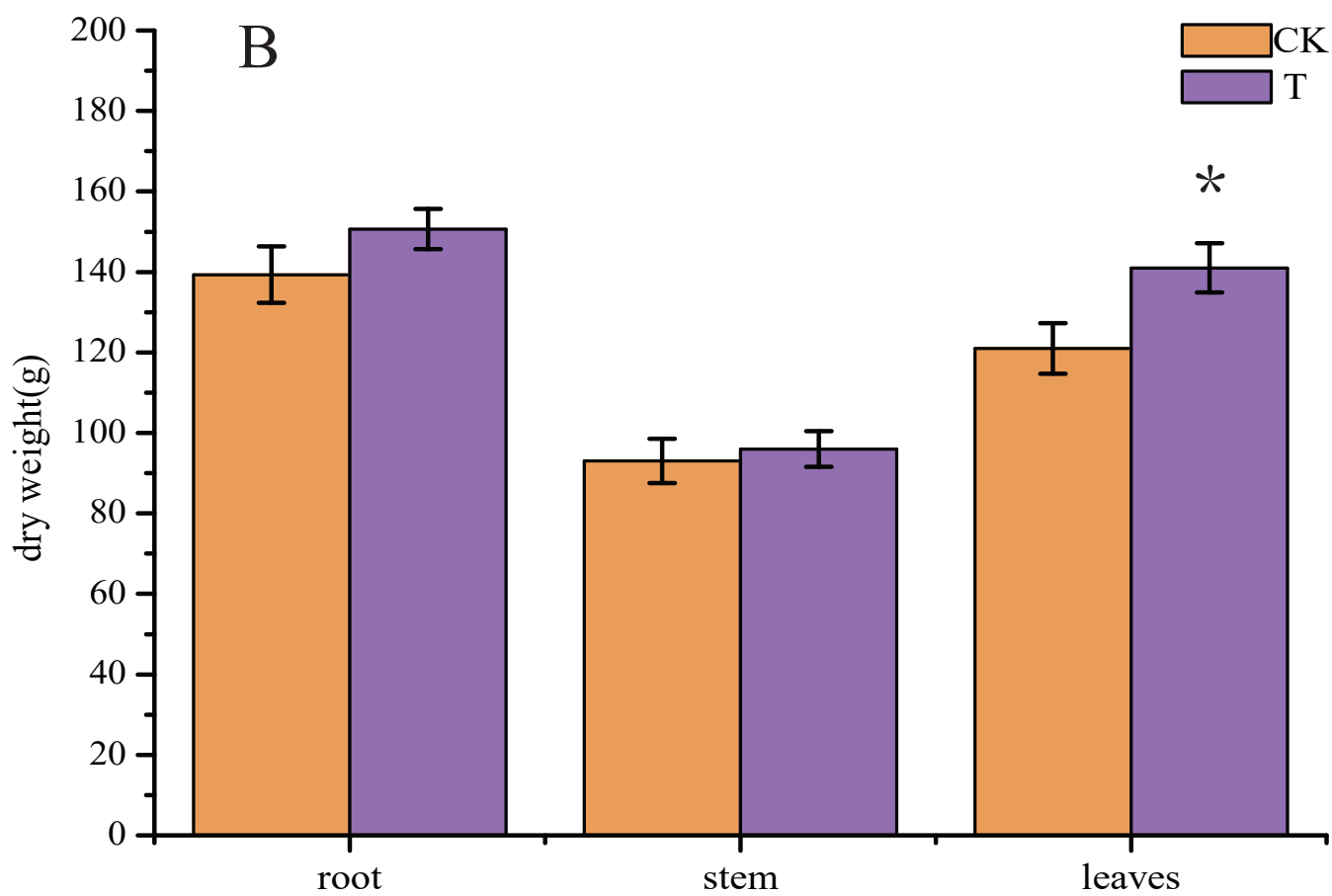

Supplement: S3 Data — (PDF) [file pone.0224556.s003.pdf]

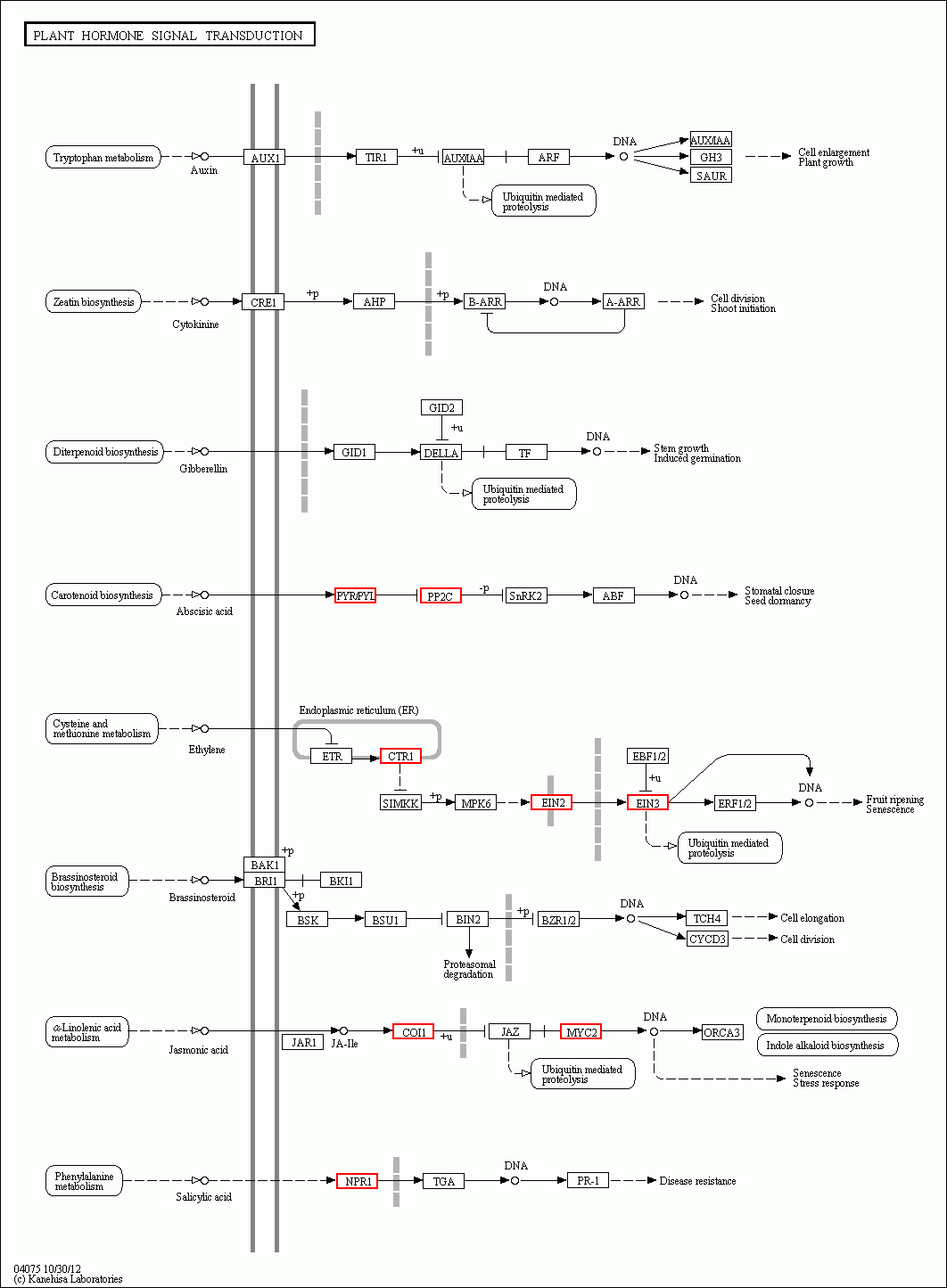

Supplement: S9 Data — (PNG) [file pone.0224556.s009.png]
