# Supplementary material for: Transcriptome sequencing reveals the effect of biochar improvement on the development of tobacco plants before and after topping
Source: PLoS One. 2019 Oct 31;14(10):e0224556. doi: 10.1371/journal.pone.0224556 (PMC6822942; doi:10.1371/journal.pone.0224556)
Supplement: S7 Data — (PDF) [file pone.0224556.s007.pdf]

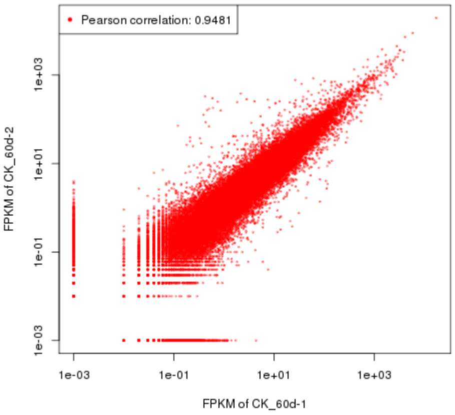

CK_60d-1-vs-CK_60d-2

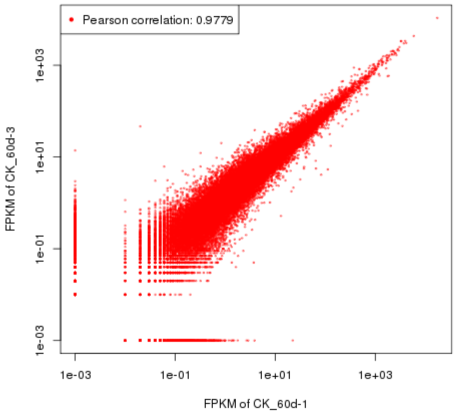

CK_60d-1-vs-CK_60d-3

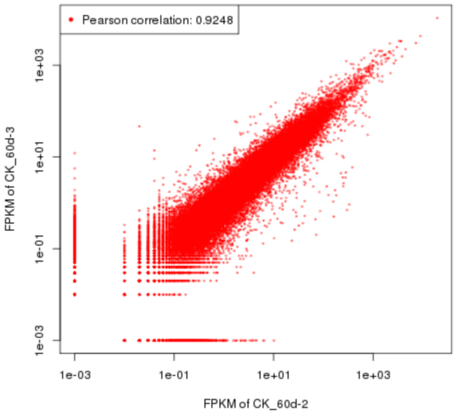

CK_60d-2-vs-CK_60d-3

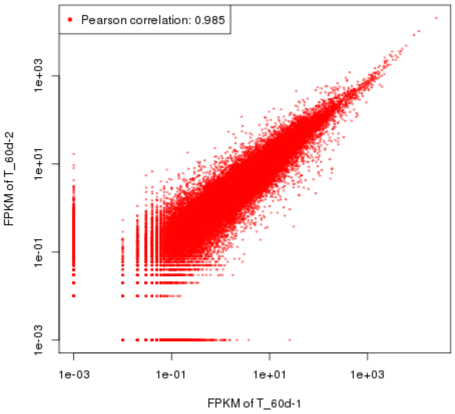

T_60d-1-vs-T_60d-2

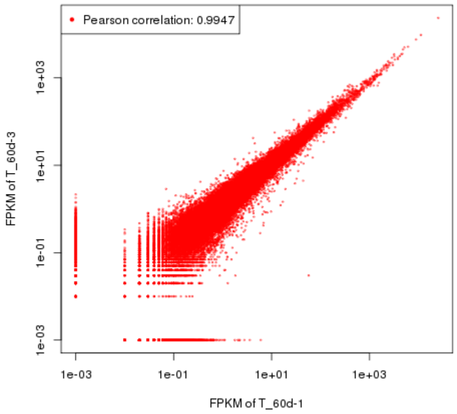

T_60d-1-vs-T_60d-3

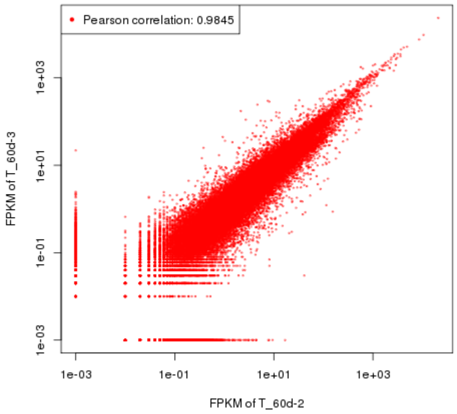

T_60d-2-vs-T_60d-3

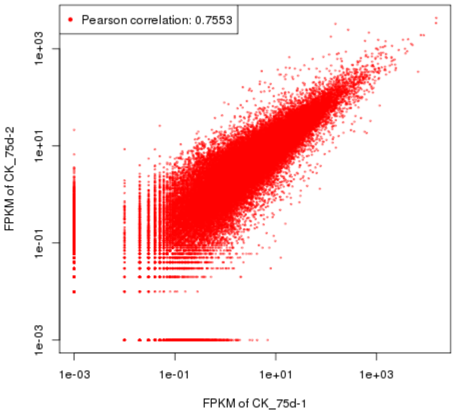

CK_75d-1-vs-CK_75d-2

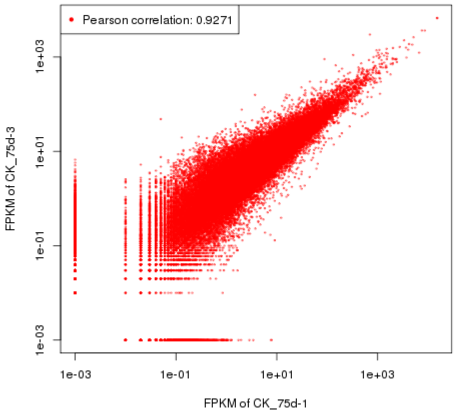

CK_75d-1-vs-CK_75d-3

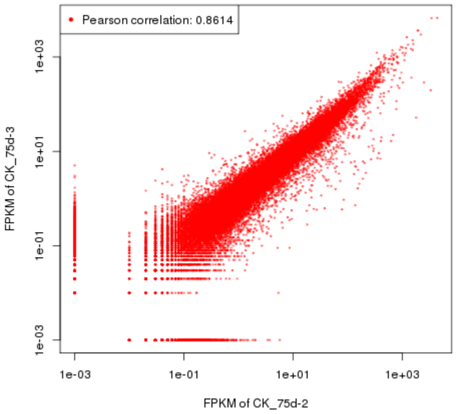

CK_75d-2-vs-CK_75d-3

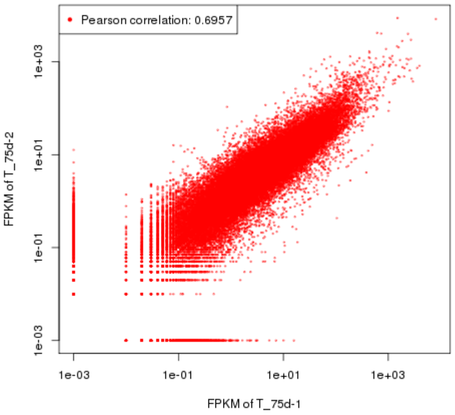

T_75d-1-vs-T_75-2

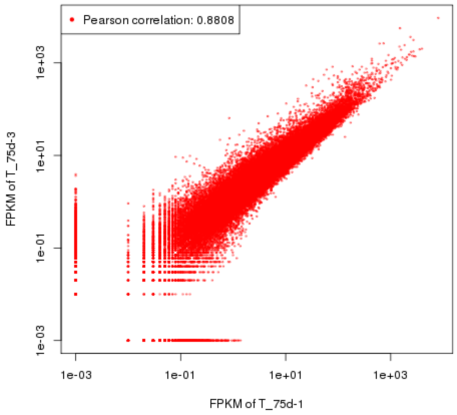

T_75d-1-vs-T_75d-3

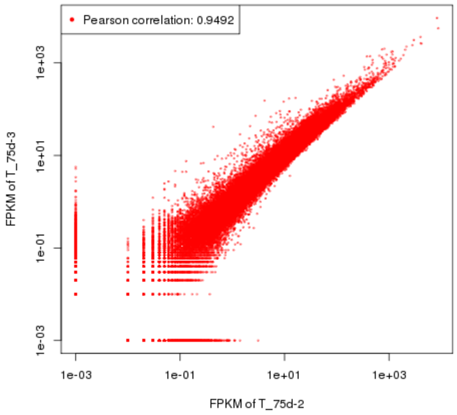

T_75d-2-vs-T_75d-3
