# Supplementary material for: Transcriptome sequencing reveals the effect of biochar improvement on the development of tobacco plants before and after topping
Source: PLoS One. 2019 Oct 31;14(10):e0224556. doi: 10.1371/journal.pone.0224556 (PMC6822942; doi:10.1371/journal.pone.0224556)
Supplement: S10 Data — (DOCX) [file pone.0224556.s010.docx]

Supplementary information 10. The correlative statistic for the gene between KEGG and qRT-PCR expressed genes

| treatment | Pearson's r | p-value |
| --- | --- | --- |
| gene20172 | 0.93 | 0.0015 |
| gene13736 | 0.45 | 0.034 |
| gene19211 | 0.81 | 0.093 |
| gene19524 | 0.87 | 0.0020 |
