# Supplementary material for: Transcriptome sequencing reveals the effect of biochar improvement on the development of tobacco plants before and after topping
Source: PLoS One. 2019 Oct 31;14(10):e0224556. doi: 10.1371/journal.pone.0224556 (PMC6822942; doi:10.1371/journal.pone.0224556)
Supplement: S5 Data — (PDF) [file pone.0224556.s005.pdf]

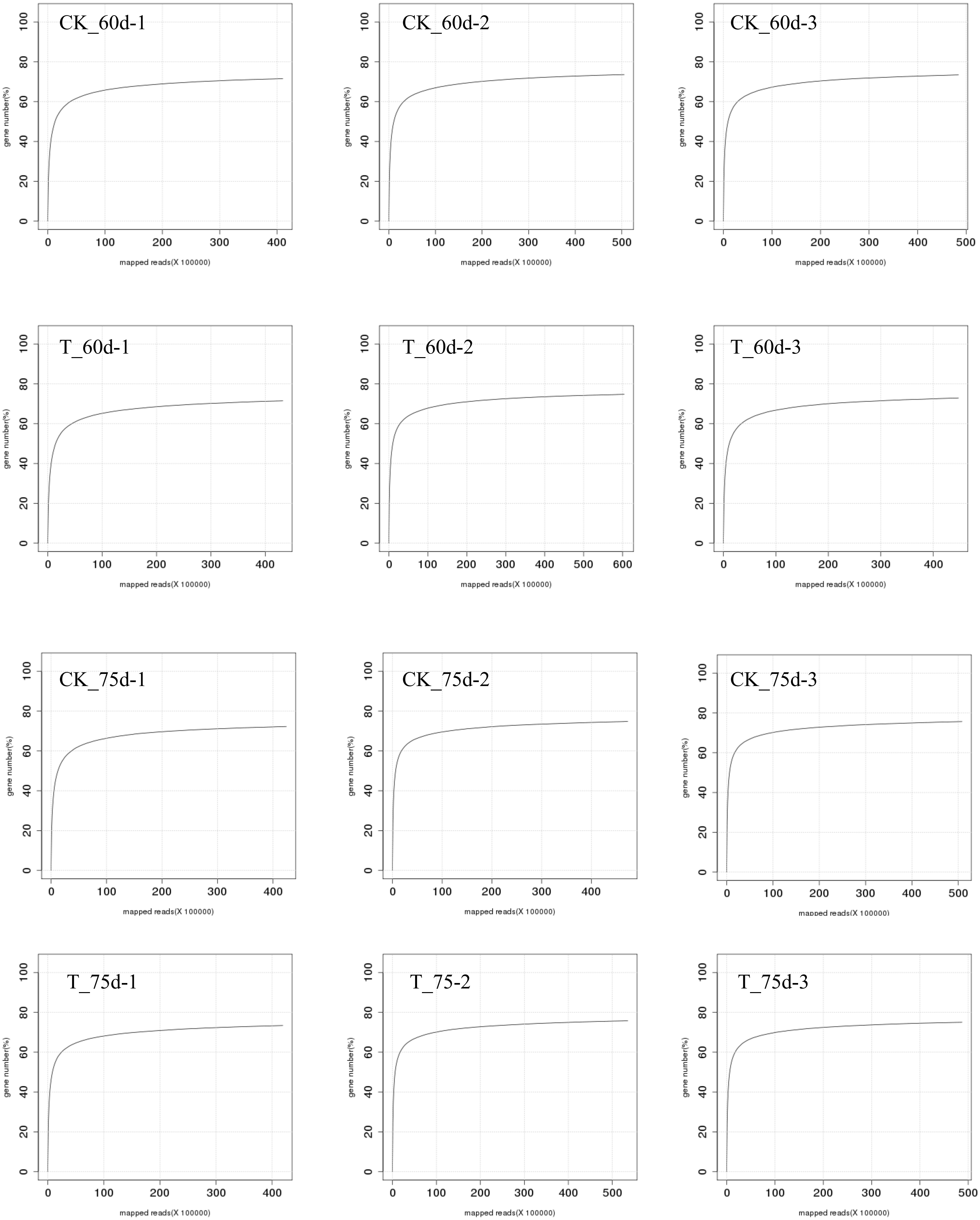

CK_60d-1
CK_60d-2
CK_60d-3
T_60d-1
T_60d-2
T_60d-3
CK_75d-1
CK_75d-2
CK_75d-3
T_75d-1
T_75-2
T_75d-3
gene number(%)
mapped reads(X 100000)
